# Translation, Cross-Cultural Adaptation, and Psychometric Properties of the Arabic Version of the MOS Pain Effect Scale in Individuals with Multiple Sclerosis

**DOI:** 10.3390/healthcare14030285

**Published:** 2026-01-23

**Authors:** Alaa M. Albishi, Zainab S. Alshammari, Sarah S. Almhawas, Dalia M. Alimam, Manal H. Alosaimi, Salman Aljarallah

**Affiliations:** 1 Department of Rehabilitation Health Sciences, College of Applied Medical Sciences, King Saud University, Riyadh 11433, Saudi Arabia; 2 Radiological Sciences Department, College of Applied Medical Sciences, King Saud University, Riyadh 11433, Saudi Arabia; 3 Department of Medicine, College of Medicine, King Saud University, Riyadh 11472, Saudi Arabia

**Keywords:** multiple sclerosis, pain, fatigue, Arabic version, validity, psychometric, quality of life

## Abstract

**Purpose**: This study aimed to translate the Pain Effects Scale (PES) into Arabic, evaluate its cultural adaptation, and assess its psychometric properties (validity and reliability) among patients with Multiple Sclerosis (MS). **Method**: The translation and cultural adaptation followed published guidelines. A total of 121 patients with MS completed the PES and several other assessments: the Short-Form McGill Pain Questionnaire (SF-MPQ), the Patient Health Questionnaire-9 (PHQ-9), the Modified Fatigue Impact Scale (MFIS), and the Multiple Sclerosis Impact Scale (MSIS-29), to evaluate construct validity. Reliability was assessed after two weeks using the intraclass correlation coefficient (ICC) and internal consistency (Cronbach’s α). **Results**: The Arabic version of Pain Effects Scale PES-Ar demonstrated good internal consistency (Cronbach’s α = 0.910) and strong test–retest reliability (ICC (2,1) = 0.88; 95% CI: 0.85–0.92). The corrected item–total correlations for all six items ranged from 0.591 to 0.840. No floor or ceiling effects were observed. Content validity indices were high (I-CVI and S-CVI = 1.00). Construct validity was supported by moderate correlations with PHQ-9 (r = 0.677), MFIS (r = 0.66), and SF-MPQ (r = 0.586), and a weak correlation with the MSIS-29. **Conclusions**: The PES-Ar showed strong validity and reliability for assessing the impact of pain in Arabic-speaking individuals with MS in Saudi Arabia.

## 1. Introduction

Multiple sclerosis (MS) is an inflammatory neurodegenerative disorder of the central nervous system (CNS), characterized by demyelination and axonal damage, often leading to lifelong disability and reduced quality of life (QOL) [1,2,3,4]. MS primarily affects young adults aged 20–40 years, with a female-to-male ratio of 3:1, and impacts approximately 2.9 million people globally [5,6,7]. In Saudi Arabia, the prevalence was recently estimated at 40.4 per 100,000 and the rates are projected to rise significantly in the country [8].

Pain is a common symptom in individuals with multiple sclerosis (MS), affecting approximately 50–65% of patients and presenting with diverse types, onset patterns, and severities [9]. According to the International Association for the Study of Pain, MS-related pain can be classified into three main categories: (1) nociceptive pain, which arises from activation of peripheral nociceptors due to tissue damage or inflammation rather than lesions of the somatosensory pathways; (2) neuropathic pain, caused by a lesion or disease affecting the somatosensory nervous system, such as MS plaques in the spinal cord, brainstem, or thalamocortical pathways; and (3) nociplastic pain, which emerges from altered nociception without clear peripheral tissue injury or somatosensory system damage and often indicates central sensitization. This “centralized” pain phenotype is characterized by widespread symptoms that can significantly impair daily functioning [10,11,12].

In a 10-year follow-up of chronic pain in people with MS, a significant decline in the quality of life was seen [13]. MS alone can cause functional problems, but the presence of pain can lead to more impairment [14]. Pain affects both the physical and emotional well-being of the person and interferes with most of their daily activities, such as sleep, work, and recreational and social activities, reducing the quality of life and leading to depression and other comorbidities [14,15,16,17,18].

Pain assessment in MS has been addressed through various instruments, including components within broader quality of life measures [16,19]. The Multiple Sclerosis Quality of Life Inventory (MSQLI) is a disease-specific health-related quality of life (HRQOL) assessment that evaluates the impact of illness and treatment on physical, emotional, and social well-being [20]. This MS inventory has been validated in a cohort of 300 MS patients [21]. and includes ten subscales, one of which is the Medical Outcomes Study Pain Effects Scale (PES) [21,22]. While abbreviated versions of MS quality of life measures, such as MSQOL-54 and MSQOL-29, have been validated and translated into various languages including Arabic [23,24], Italian [25], French Canadian [26], Turkish [27], Persian [28,29], Hungarian [30], Serbian [31], Spanish [32], Indonesian [33], and Bosnian [34], these instruments include only limited pain assessment items (2–3 questions) derived from the original PES, providing insufficient detail for comprehensive pain evaluation.

The PES measures pain severity and its impact on daily activities [35]. PES has 6 items related to mood, walking or moving around, sleep, working (outside the home and at home), recreational activities, and enjoying life in the past 4 weeks [36]. Responses indicate the degree of pain interference, with responses ranging from 1 (not at all) to 5 (to an extreme degree) [36]. Scores range from 6 to 30, with higher scores indicating greater pain interference [36]. Although the PES has demonstrated excellent validity and reliability in various medical and MS cohorts [22,37], the full 6-item scale has not yet been translated nor validated into the Arabic language.

The need for a comprehensive Arabic version of the PES is particularly important given that existing Arabic-validated instruments like MSQOL-29-Ar include only basic pain interference questions (focusing on work and pain severity), while the complete PES addresses additional crucial domains such as mood, sleep, recreational activities, and life enjoyment. This comprehensive assessment is essential for understanding the full impact of pain on daily functioning in Arabic-speaking patients with MS. Thus, this study aims to translate the PES scale into Arabic, evaluate its cultural adaptation, and assess its psychometric properties (validity and reliability) among patients with MS.

## 2. Materials and Methods

### 2.1. Study Design

A cross-cultural adaptation and psychometric measurement validation study was conducted using a convenience sampling method to test a newly translated and cross-culturally adapted Arabic version of PES. This study comprised two parts: (a) the translation and cross-cultural adaptation process of the PES scale and (b) the validation of the scale in the Arabic language.

### 2.2. Ethical Considerations

This study was ethically approved by the Institutional Review Board (IRB) at King Saud University (No. E-24-9395). A written consent was obtained from all participants before participation in this study. In addition, permission was granted by the main developers of the PES scale to translate it into the Arabic language and use it in this research project.

### 2.3. Sample

The study was conducted on 121 patients with MS from King Khalid University Hospital (KKUH), Riyadh, Kingdom of Saudi Arabia, between January and May 2025. In this study, a participant of either gender was recruited if the individual was (1) 18 years and above [38,39] and (2) clinically diagnosed with MS. We excluded participants who (1) scored less than 26 on the Montreal Cognitive Assessment (MOCA) test (a screening tool that is used to detect patients with cognitive impairment) [40] or (2) were unable to read or speak Arabic [40].

### 2.4. Procedures

#### 2.4.1. Phase 1: Translation and Cross-Cultural Adaptation Process

The Translation and cross-cultural adaptation of the original PES version into Arabic were conducted in accordance with the guidelines outlined by Beaton [41] to ensure conceptual equivalence between the original and translated versions. The process was conducted in the following stages:

1. Forward and Back-Translation: Initially, two forward translations (T1 and T2) of the English PES into Modern Standard Arabic (MSA) were conducted by two independent bilingual translators (both fluent in English and Arabic), one with a medical background and the other without. These forward translations (T1 and T2) were subsequently consolidated into a single Arabic version (T-12) by a professional translator and a medical specialist to address any discrepancies. The synthesized Arabic version was then subjected to a back-translation process into English, where two independent bilingual translators, who had no prior knowledge of the PES, translated it back into English to produce two comparative versions (BT1 and BT2).
2. Expert Committee Review: In line with COSMIN guidelines, once the translation process was completed, the Arabic version of Pain Effects Scale (PES-Ar) was evaluated by a panel of ten independent experts, consisting of ten healthcare professionals, including methodologists, medical faculty, neurorehabilitation clinicians, and linguistics and translation specialists. The committee reviewed both the English and Arabic versions to assess face and content validity of each item individually and produced the pre-final version of the PES-Ar. The clarity and relevance of each item were assessed using two separate four-point Likert scales: one for relevance (1 = not relevant, 2 = somewhat relevant, 3 = quite relevant, 4 = highly relevant), and another for clarity (1 = not clear, 2 = item needs some revision, 3 = clear but needs minor revision, 4 = very clear). Experts were asked to assign a score from 1 to 4 for each item on both dimensions. For the relevance assessment, scores of 3 or 4 were recorded as 1 (indicating acceptable relevance), and scores of 1 or 2 were recorded as 0. Based on these ratings, the Content Validity Index (CVI) for each item (I-CVI) was computed by dividing the number of experts who rated the item as 3 or 4 by the total number of experts [42]. The Scale-Level CVI (S-CVI) was also calculated to reflect the overall content validity of the instrument [43]. To attain satisfactory content validity, an S-CVI/Avg score of at least 0.83 is required [44]. The committee ensured that conceptual equivalence was maintained and that the items were culturally relevant to the target population.
3. Cognitive Debriefing and Pilot Testing: To obtain qualitative feedback, a pilot study was conducted with 20 individuals with MS using a structured cognitive debriefing approach. Face-to-face interviews were carried out, during which probing questions were used to assess participants’ understanding of each item (e.g., “In your own words, what does this item ask?”). Participants were also invited to share their experiences and perceptions of the current version of the questionnaire, and all feedback was systematically recorded. Based on the findings of this process, the PES-Ar was finalized.
4. Cultural Adaptation: Based on expert feedback and pilot testing, culturally specific expressions were adjusted (culturally adapted) to ensure appropriate understanding.

#### 2.4.2. Phase 2: Validity and Reliability Process

Once the final PES-Ar was established, its psychometric properties were assessed, including internal consistency, test–retest reliability, measurement error, floor and ceiling effects, and validity. Data collection included demographic information such as age, gender, educational level, employment status, and marital status. The responsible clinician obtained clinical data on the diagnosed condition subtype and disease duration from each patient’s medical records.

During the first visit, the patients with MS were asked to complete five Arabic self-report questionnaires: the Short-form McGill Pain Questionnaire (SF-MPQ), the Patient Health Questionnaire-9 (PHQ-9), Modified Fatigue Impact Scale (MFIS), Multiple Sclerosis Impact Scale (MSIS-29) and the PES-Ar. After two weeks (T2), participants were asked to complete the PES-Ar in the second visit to examine the test–retest reliability, two-week interval was selected for test–retest reliability, as it is widely recognized as an appropriate timeframe to balance the prevention of recall bias with the maintenance of clinical stability [45]. Additionally, participants rated any perceived change in their health status using the Global Rating of Change (GROC) during the second visit to determine whether their health condition had changed between the two assessments.

### 2.5. Outcome Measures

#### 2.5.1. Pain Effects Scale

The Pain Effects Scale is a six-item questionnaire that evaluates how pain and unpleasant sensations affect mood, mobility, sleep, work, leisure activities, and enjoyment of life [46]. Participants indicate how much pain affected these areas of functioning over the previous four weeks on a scale of 1 (not at all) to 5 (to a severe degree) [46]. The PES total score can be calculated by summing the ratings for each of the six items, which range from 6 to 30 [46]. Higher scores indicate more pain interference [46]. PES has been validated and shown great internal consistency in a large medical sample (α = 0.92) for the original version [37,46].

#### 2.5.2. The Arabic Version of the Short-Form McGill Pain Questionnaire (SF-MPQ)

The SF-MPQ is a 15-item questionnaire that evaluates the emotional and sensory aspects of pain [47]. The fifteen items are scored on a four-point level of pain scale (0 = none, 1 = mild, 2 = moderate, and 3 = severe) [47]. To calculate pain intensity, add the intensity values for each descriptor: sensory (11 items), emotional (4 items), and total (15 items) pain scores [47]. To evaluate the total pain experience, two items are added to the 15-item questionnaire [47]. A visual analog scale is also included in the SF-MPQ [47]. The SF-MPQ was reliable and valid among Arabic-speaking patients with good test–retest reliability by intraclass correlation coefficient (ICC = 0.71) and internal consistency (Cronbach’s α  =  0.85) [47].

#### 2.5.3. The Arabic Version of the Patient Health Questionnaire-9 (PHQ-9)

The PHQ-9 is a nine-item self-administered instrument used to assess the severity of depressive symptoms based on criteria outlined in the Diagnostic and Statistical Manual of Mental Disorders (DSM-IV) [48]. Participants rate how frequently they experienced each symptom during the preceding two weeks using a four-point response format ranging from 0 (not at all) to 3 (nearly every day) [48]. Individual item scores are summed to yield a total score between 0 and 27, with higher scores indicating greater depressive symptom severity. A threshold score of 10 or higher is commonly applied to identify individuals at risk for major depressive disorder [49]. The Arabic version of the PHQ-9 has demonstrated adequate psychometric properties, including good internal consistency (Cronbach’s α = 0.826) and strong test–retest reliability (ICC = 0.886) [49,50,51].

#### 2.5.4. The Arabic Version of the Modified Fatigue Impact Scale (MFIS)

Fatigue was evaluated using the MFIS, a multidimensional self-report scale originally adapted from the Fatigue Impact Scale [52]. The MFIS consists of 21 items designed to capture the impact of fatigue on physical, cognitive, and psychosocial functioning [52]. Responses are scored on a five-point Likert-type scale ranging from 0 (never) to 4 (almost always) [52]. Total scores range from 0 to 84, with higher values reflecting a greater perceived impact of fatigue. The Arabic adaptation of the MFIS is valid and reliable, exhibiting excellent internal consistency (Cronbach’s α = 0.968) and high test–retest reliability (ICC = 0.920) [52].

#### 2.5.5. The Arabic Version of the Multiple Sclerosis Impact Scale (MSIS-29)

The MSIS-29 is developed as a disease-specific measure of HRQOL. It is a self-report questionnaire completed by the patient using the patient’s perspective on disease impact in MS [38,53]. Consisting of 29 items, the measurements and two subscales are physical (20 items) and psychological (9 items) [38]. All items have a Likert-type response format (“Not at all,” “A little,” “Moderately,” “Quite a lot”, and “Extremely”) to assess the level of difficulty experienced by individuals in the last two weeks [38]. Scores on the individual items are added and then transformed to a 0–100 scale, thereby generating two summary scores (for physical and psychological impact) [38]. The MSIS-29 was reliable and valid for use among Arabic-speaking patients with good test–retest reliability (ICC = 0.97) and excellent internal consistency (Cronbach’s α  =  0.85–0.95) [38].

#### 2.5.6. The Global Rating of Change (GROC)

The GROC is a self-report outcome measure designed to assess a patient’s perception of health status changes over time, helping to determine whether their condition has improved, worsened, or remained the same [54]. The clinician asks the patient to rate the change in their condition between an initial assessment and a follow-up visit. The GROC scale consists of 11 points, ranging from −5 to +5, where −5 represents a significant worsening, +5 indicates a significant improvement, and 0 reflects no change in the patient’s condition [54,55]. For test–retest reliability, patients with GROC scores between −2 and +2 are considered to have experienced no significant change (stable) [54]. Previous studies have confirmed that the GROC is a reliable and valid tool for measuring changes or stability in a patient’s condition [54]. The test–retest reliability showed a high (ICC = 0.90) (95% CI: 0.84 to 0.93) [54]. The scoring direction of all outcome measures is summarized in Appendix A.

### 2.6. Statistical Analysis

Data analysis was conducted using SPSS version 26 (IBM Corp., Armonk, NY, USA). Descriptive statistics (mean, standard deviation, and count) were used to describe the participants’ characteristics. The Kolmogorov–Smirnov (KS) test was used to assess the normality of data distribution. A *p*-value of less than 0.05 was considered indicative of a non-normal distribution.

#### 2.6.1. Floor and Ceiling Effect

Floor and ceiling effects. A floor or ceiling effect was considered present if more than 15% of the respondents achieved the lowest or highest possible score [45].

#### 2.6.2. Test–Retest Reliability and Internal Consistency

Internal consistency was evaluated using Cronbach’s alpha, and values between 0.70 and 0.95 were found, indicating sufficient internal consistency [45]. Item-level internal consistency was further examined by calculating corrected item–total correlations, with values ≥ 0.30 considered acceptable indicators of adequate item discrimination, as well as Cronbach’s alpha if item deleted to assess the contribution of individual items to the overall scale reliability [45].

The degree of absolute agreement between the test scores was measured using a two-way random-effect intra-class correlation coefficient model with a single rater, ICC (2,1). We use the standard error of measurement (SEM) associated with the PES-Ar to examine the measurement error. The SEM was calculated using the formula SEM = SD × √ (1 − ICC), where SD is the pooled standard deviation at baseline and ICC is the test–retest intraclass correlation coefficient [56]. We also used the minimal detectable change with 95% confidence (MDC95), which was used to quantify the true change in the PES-Ar beyond the measurement error. The MDC95 was calculated using the following formula: MDC95 = SEM × 1.96 × √2 [56].

#### 2.6.3. Content, Structural and Construct Validity

Content validity was assessed through expert evaluation of the instrument’s items. An Item-Level Content Validity Index (I-CVI) value of 0.78 or higher is generally considered acceptable [43], while a Scale-Level Content Validity Index (S-CVI) value of 0.90 or above is typically interpreted as evidence of strong content validity [57].

To evaluate the structural validity of the PES-Ar, an exploratory factor analysis (EFA) was conducted using principal axis factoring (PAF). The Kaiser–Meyer–Olkin (KMO) measure and Bartlett’s test of sphericity were used to assess sampling adequacy and data suitability for factor analysis. The number of factors were determined based on eigenvalues greater than one and visual inspection of the scree plot.

The construct validity of the PES-Ar was assessed by examining its correlations with conceptually related outcome measures, including the PHQ-9, SF-MPQ, MFIS, and the MSIS-29 subscales. Spearman’s rank correlation coefficient was used to determine the strength and direction of these associations. Correlation coefficients between 0.1 and 0.4 were considered weak, those between 0.4 and 0.7 as moderate, and values above 0.7 as strong [45,58]. Construct validity was deemed supported when at least 75% of the predefined hypotheses were confirmed [45,58].

Due to the limited number of studies evaluating the construct validity of the Pain Effects Scale (PES) with external outcome measures, predefined hypotheses were developed based on established clinical and empirical evidence linking pain interference to key symptom domains in individuals with multiple sclerosis (MS). The expected associations were: (1) a moderate to strong positive correlation between the PES-Ar and the PHQ-9, reflecting the well-known relationship between pain interference and depressive symptoms in MS [59]; (2) a moderate to strong positive correlation with the SF-MPQ, as both measure pain-related experiences; (3) a moderate to strong positive correlation with the MFIS, given the co-occurrence of pain and fatigue in MS [59]; (4) a weak to moderate positive correlation with the MSIS-29 physical subscale, reflecting partial conceptual overlap in activity-level impacts [60]; and (5) a weak positive correlation with the MSIS-29 psychological subscale, which captures broader psychological constructs beyond pain interference [61]. These predefined hypotheses formed the basis for evaluating construct validity.

The selection of comparator instruments was guided by their theoretical relevance to pain interference and the availability of validated Arabic versions. The PHQ-9 and MFIS were selected to assess associations with depression and fatigue, both of which are commonly linked to pain in individuals with MS. The SF-MPQ was included to examine correlations with pain intensity and sensory–affective pain experiences. The MSIS-29 subscales were chosen to explore relationships between pain interference and the broader physical and psychological impacts of MS. Other MS-specific quality of life instruments, such as the MSQOL-54, were not included because they measure overall health-related quality of life and only incorporate pain as a minor subdomain, making them less suitable for evaluating the construct validity of a pain interference–specific measure. Accordingly, the selected instruments were considered the most appropriate comparators for this analysis.

#### 2.6.4. Sample Size Estimation

A minimum of 50 participants is recommended for evaluating measurement properties, according to the COSMIN (COnsensus-based Standards for the selection of health Measurement Instruments) guidelines [45,56]. However, it has also been suggested that a sample size of at least 100 would be more appropriate [56]. For Test–retest reliability, a minimum of 30 participants is considered acceptable, while a sample size of 50 or more is required to achieve high reliability [56]. Our study included 121 patients with MS, and 50 of them who responded were used in the test–retest reliability analysis.

## 3. Results

### 3.1. Phase 1: Translation and Cross-Cultural Adaptation

After the translation process, based on the feedback of experts and patients, certain words were modified to enhance clarity and improve patient comprehension. Minor adjustments were made to ensure wording clarity and relatability for patients. For example, “mood” was translated as “your mood”, “outside or inside the home” as “from home or outside”, “recreational activities” as “your recreational activities”, and “enjoyment of life” as “your ability to enjoy your life”.

The pre-final version of the PES-Ar was administered to a group of 20 individuals with MS, including 8 males and 12 females. These participants were not included in the primary study phase evaluating the instrument’s measurement properties. All participants completed the questionnaire without any issues. Additionally, their comprehension of the items was assessed to ensure clarity and relatability. All items were clear and easy to understand for MS patients. The final PES-Ar items with the rationale for each modification are documented in the Appendix A.

#### 3.1.1. Demographic Data and Characteristics of the Participants

Table 1 presents the demographic and clinical characteristics of the study participants at baseline (*n* = 121) and the retest subsample (*n* = 50). At baseline, the mean age of participants was 37.6 ± 7.75 years, and the majority were female (59.5%). The mean disease duration was 13.08 ± 5.34 years, and most participants had the relapsing-remitting (RR) type of MS (86.78%), while 13.22% had the secondary-progressive (SP) type. Additionally, 27.3% of participants reported a prior diagnosis of depression. The retest subsample included 50 participants from the total, comprising 30 women and 20 men. The mean age in the retest group was 38.8 ± 6.9 years, and the mean disease duration was 13.2 ± 5.8 years. In this subsample, 84% of participants had RR of MS, and 16% had SP of MS. A prior diagnosis of depression was reported by 22% of the participants who retested.

#### 3.1.2. Floor and Ceiling Effects

Floor and ceiling effects were assessed using the full sample (*n* = 121). For the total PES-Ar score, 15 participants (12.4%) achieved the lowest possible score and one participant (0.8%) achieved the highest possible score, indicating no floor or ceiling effects at the scale level (>15%). At the item level, floor effects exceeding 15% were observed across several items, whereas no ceiling effects were detected (Table 2).

### 3.2. Phase 2: Validity and Reliability Process

#### 3.2.1. Internal Consistency and Test–Retest Reliability

The results of the internal consistency analysis for the 121 respondents indicated excellent reliability, with Cronbach’s alpha of 0.910 for the PES-Ar. The corrected item–total correlations for all six items ranged from 0.591 to 0.840, exceeding the recommended threshold of 0.30. Furthermore, the “alpha if item deleted” values (0.881–0.914) indicated that removing any item would not meaningfully improve the scale’s internal consistency (Table 3).

To assess the stability of the scale over time, a test–retest reliability analysis was conducted with 50 participants who completed the PES-Ar on two occasions, two weeks apart. All 50 participants reported no change between the two assessments based on the GROC scale; therefore, their data were used to calculate the intraclass correlation coefficient (ICC). The PES-Ar demonstrated excellent test–retest reliability, with an ICC (2,1) of 0.88 (95% confidence interval: 0.85–0.92), as shown in (Table 4).

#### 3.2.2. Measurement Error

Based on the ICC reliability coefficient, the SEM of the PES-Ar was 2.00, while the MDC95 was 5.54 (Table 4).

#### 3.2.3. Validity Process

##### Face, Content Validity

For content validity, ten experts evaluated the PES-Ar and agreed that all items were appropriate. The item-level content validity index (I-CVI) for all items was 1.00, and the scale-level content validity index (S-CVI) was also 1.00, indicating excellent content validity. Regarding face validity, patients did not report any difficulties understanding or completing the questionnaire, suggesting that the items were clear and appropriate for the target population.

##### Structural Validity

**Figure 1:**
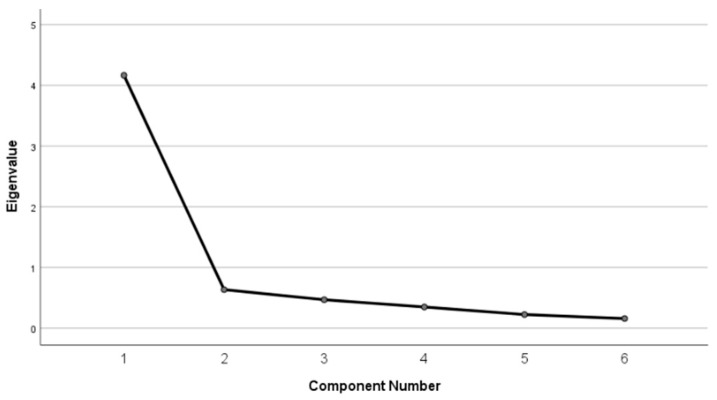
Scree plot from exploratory factor analysis of PES-Ar.

To evaluate the structural validity of the PES-Ar, an EFA was conducted using PAF. The KMO measure demonstrated excellent sampling adequacy (KMO = 0.89), and Bartlett’s test of sphericity was significant (χ^2^ = 488.85, df = 15, *p* < 0.001), confirming that the data were suitable for factor analysis. A single-factor solution was identified based on eigenvalues greater than one and visual inspection of the scree plot, which revealed a distinct inflection after the first factor (Figure 1). This factor explained 69.4% of the total variance. All six items loaded strongly on the single factor, with loadings ranging from 0.69 to 0.90 (Table 5), supporting the unidimensional structure of the PES-Ar.

##### Construct Validity

The construct validity of the PES-Ar was evaluated by testing predefined hypotheses about the expected strength of correlations with related outcome measures. Spearman’s rank-order correlation coefficients were calculated to examine the relationships between the PES-Ar and the PHQ-9, SF-MPQ, MFIS, and MSIS-29 subscales. The correlation results are summarized in (Table 6).

Moderate positive correlations were observed between the PES-Ar and the PHQ-9 (r = 0.677, *p* < 0.001), MFIS (r = 0.660, *p* < 0.001), and SF-MPQ (r = 0.586, *p* < 0.001), supporting the construct validity of the PES-Ar and aligning with the predefined hypotheses for related constructs. A weak but statistically significant correlation was found between the PES-Ar and the MSIS-29 physical subscale (r = 0.188, *p* = 0.039), reflecting partial conceptual overlap at the activity level while suggesting that the instruments assess related but non-identical constructs. In contrast, the correlation between the PES-Ar and the MSIS-29 psychological subscale was weak and not statistically significant (r = 0.085, *p* = 0.354), indicating that pain interference is not strongly associated with broader psychological impacts of multiple sclerosis. Overall, four of the five predefined hypotheses (80%) were confirmed, providing evidence in support of the construct validity of the PES-Ar.

## 4. Discussion

From a pain mechanism perspective, pain in MS can arise from nociceptive, neuropathic, and nociplastic processes, which often coexist and interact. Although the PES measures pain ‘interference’ rather than classifying specific pain mechanisms, its emphasis on functional and psychosocial effects makes it a robust tool for evaluating the overall burden of pain in MS, especially in cases where mixed pain types are common.

Translating standardized measures into participants’ native languages enhances usability and ensures accurate and culturally relevant data collection. However, there remains a critical gap in comprehensive pain assessment tools for Arabic-speaking patients with MS. Existing Arabic instruments, such as the MSQOL-29-Ar and MSQOL-54, offer limited pain assessment, typically using only 2–3 items focused on severity and work interference [23,24].

These abbreviated assessments, while useful for general screening, do not capture the multifaceted impact of pain on various life domains that the complete PES addresses, including effects on mood, sleep quality, recreational activities, and overall life enjoyment.

Therefore, this study aimed to cross-culturally adapt the PES into Arabic and to evaluate its psychometric properties, including validity (face, content, structural and construct), reliability (internal consistency and test–retest), measurement error, and floor/ceiling effects, among people with MS.

Content validity is a relevant measure that determines whether the instrument measures what it was designed to measure [42,43]. To assess the scale’s content validity, both the I-CVI and the S-CVI were calculated. Based on prior research, an I-CVI of 0.78 or higher and an S-CVI of 0.90 or above are generally regarded as acceptable [42]. In this study, the I-CVI and S-CVI were both 1.00, indicating that the expert panel unanimously agreed on the clarity and relevance of the Arabic version of the PES-Ar. Also, the content validation process involved a comprehensive review by an expert committee, which assessed various aspects such as linguistic clarity and cultural relevance before finalizing the pre-test version. The PES-Ar was subsequently pilot-tested with 20 individuals with MS, who reported the scale to be clear, relevant, and easy to complete, with no difficulties encountered. As emphasized by Yusoff (2019) [44], establishing face and content validity is essential to support the validity of an assessment tool, such as questionnaires, especially for research purposes.

The PES-Ar scores range from 6 to 30, with higher scores indicating a greater effect of pain on patients with MS. Floor and ceiling effects are considered present when more than 15% of participants obtain the lowest or highest possible score on the questionnaire [54]. In our study, no floor or ceiling effects were observed at the scale level.

The internal consistency of the PES-Ar in this study was good, with an overall Cronbach’s alpha of 0.910. These results are consistent with those of the original instrument (alpha range: 0.770–0.960) [46]. Regarding the reliability of the PES-Ar, as measured by ICCs, the instrument also demonstrated high test–retest reliability ICC (2,1) = 0.88; 95% confidence interval (0.85, 0.92) within a two-week interval in the current study. These findings are comparable to the reliability reported for the Arabic version of the MSQOL-29 (MSQOL-29-Ar) [24]. The pain item, which is part of the Mental Health Composite (MHC), demonstrated strong reliability, showed an internal consistency of 0.87, and the test–retest reliability ICC (2,1) was 0.86 (95% CI: 0.77–0.91), with a test–retest (r) of 0.75 [24]. The high corrected item–total correlations (all items > 0.30) and the lack of improvement in Cronbach’s alpha following item deletion confirm that all six items are essential and contribute meaningfully to the scale’s reliability, supporting the retention of the full PES-Ar.

Exploratory factor analysis supported a unidimensional structure of the PES-Ar, indicating that the six items assess a single underlying construct of pain interference. This finding supports the structural validity of the Arabic version for use in individuals with MS.While exploratory methods were sufficient to identify the underlying structure of the scale, future studies employing confirmatory factor analysis (CFA) and ordinal-based estimation methods, such as polychoric correlations, may further strengthen these findings by accounting for the categorical nature of Likert-scale items. This is particularly relevant, as ordinal methods can provide more accurate estimates of factor loadings and reliability when data deviate from multivariate normality [62]. Regarding measurement error, it refers to the random and systematic variation in a patient’s score that does not reflect actual changes in the construct being assessed [63]. The SEM quantifies the amount of variation in repeated scores due to measurement error, serving as the standard deviation associated with a single observed score [63]. In this study, the SEM for the PES-Ar was estimated at 2.00. The MDC represents the smallest change in a score that can be interpreted as a true change exceeding measurement error [64]. The MDC95, which reflects a conservative threshold for detecting real change, was calculated as 5.54. These findings indicate that the PES-Ar is capable of detecting changes beyond measurement error in pain-related participation, supporting its use for monitoring change over time in both research and clinical settings. From a clinical perspective, an MDC95 value of 5.54 may aid clinicians and researchers in distinguishing true score changes from measurement error when evaluating changes over time, such as following interventions; however, it alone does not determine clinical importance.

The construct validity of the PES-Ar was examined by examining its relationships with measures that evaluate theoretically related constructs, including fatigue, depressive symptoms, pain intensity, and the physical and psychological effects of MS. Moderate positive correlations were observed between the PES-Ar and the MFIS, PHQ-9, and SF-MPQ, which support the construct validity of the PES-Ar. These findings are consistent with previous studies demonstrating close relationships between pain interference, fatigue, depressive symptoms, and pain intensity [65,66,67,68,69,70].

In particular, the moderate link between the PES-Ar and fatigue aligns with previous evidence suggesting that pain and fatigue frequently co-occur in individuals with MS and may share common underlying mechanisms within the central nervous system [65,66,67,68]. One proposed model suggests that pain could influence fatigue both directly and indirectly, with sleep disturbance acting as a potential mediating factor [65].

The PES-Ar also demonstrated a significant association with depressive symptoms as measured by the PHQ-9, consistent with previous findings reporting moderate to strong relationships between pain interference and depression severity [46,69]. Similarly, the moderate correlation observed with the SF-MPQ supports the conceptual link between pain interference and pain intensity, as previously reported in validation studies of pain-related outcome measures [47].

Although a positive association between pain interference and psychological impact was hypothesized, the correlation between the PES-Ar and the MSIS-29 psychological subscale was weak and not statistically significant. This suggests that pain interference is only one part of the broader psychological impact experienced by people with multiple sclerosis. The MSIS-29 psychological subscale captures a wide range of psychological domains, including cognition, emotional well-being, fatigue, and depressive symptoms, while the PES-Ar specifically measures how much pain interferes with certain aspects of daily life. This distinction likely explains the limited strength of the observed association and aligns with evidence showing that psychological impact in MS is more strongly influenced by fatigue, cognitive impairment, and depressive symptoms than by pain interference alone [61,70].

A weak but statistically significant association was observed between the PES-Ar and the MSIS-29 physical subscale. This finding likely reflects partial conceptual overlap at the activity level, as both instruments include aspects related to physical functioning while differing in their primary constructs. Previous studies have shown that pain severity is only weakly related to physical disability in MS, supporting the distinction between pain interference and physical functioning [60].

Several limitations of this study should be acknowledged. First, the generalizability of the findings is limited due to single-center recruitment and the predominance of participants with relapsing-remitting MS. This may limit the applicability of the results to patients with other MS subtypes or those managed in different clinical settings. Second, construct validity in this study was assessed using only depression, fatigue, and the physical and psychological impact of MS. While these constructs are relevant, future studies should consider additional domains, such as health-related quality of life, to provide a more comprehensive validation of the PES-Ar. Furthermore, measurement invariance across different subgroups, such as sex, disease subtypes, or depression status, was not examined. This remains an important missing psychometric property that should be addressed in future research with larger sample sizes. Although the MDC95 provides a threshold for distinguishing true score changes from measurement error, it does not indicate whether such changes are clinically important. Because minimal important change (MIC) was not estimated in this study, the clinical interpretability of change scores remains limited and should be examined in future research. Lastly, all participants were recruited from Saudi Arabia, which may limit the generalizability of the findings to other cultures. Therefore, further multi-center research including Arabic-speaking individuals from diverse countries and cultural contexts is recommended to enhance the cross-cultural applicability of the PES-Ar.

## 5. Conclusions

The Arabic version of the PES-Ar demonstrated robust validity and reliability in assessing the comprehensive impact of pain among individuals with MS within a Saudi sample. Unlike existing Arabic pain assessment tools that provide limited evaluation, the PES-Ar offers a detailed assessment across six key domains of pain interference. The scale is easy to administer, suitable for research purposes, and clinically applicable, making it a valuable complement to existing Arabic MS assessment instruments and a practical tool for routine use in clinical settings. The findings of this study lay the foundation for advancing research and driving clinical initiatives aimed at comprehensive pain assessment among individuals with MS in Arabic-speaking populations.

## Figures and Tables

**Table 1:** Demographic and Clinical Characteristics of the Baseline (*n* = 121) and Retest (*n* = 50) Samples.

Variables	Mean ± SD or Count (%)		
		Baseline (*n* = 121)	Retest (*n* = 50)
Age (Years)		37.60 ± 7.759	38.8 ± 6.9
Sex	Male	49 (40.5%)	20 (40%)
	Female	72 (59.5%)	30 (60%)
Disease Duration (Years)		13.08 ± 5.346	13.2 ± 5.8
Disease Type	RR	105 (86.78%)	42 (84%)
	SP	16 (13.22%)	8 (16%)
Diagnose with Depression:	Yes	33 (27.3%)	11 (22%)
	No	88 (72%)	39 (78%)

RR: Relapsing-Remitting; SP: Secondary Progressive.

**Table 2:** Floor and Ceiling Effects for the PES-Ar (*n* = 121).

Scale/Item	Floor (*n*)	Floor (%)	Ceiling (*n*)	Ceiling (%)
Total PES-Ar Score	15	12.4	1	0.8
Mood	26	21.5	4	3.3
Ability To Walk or Move Around	50	41.3	11	9.1
Sleep	46	38.0	12	9.9
Normal Work	48	39.7	5	4.1
Recreational Activities	47	38.8	10	8.3
Enjoyment of Life	45	37.2	11	9.1

**Table 3:** Corrected item-total correlations and Cronbach’s alpha for PES-AR items(*n* = 121).

Items	Corrected Item-Total Correlation	Cronbach’s Alpha If Item Deleted
Mood	0.591	0.914
Ability to walk or move around	0.575	0.905
Sleep	0.755	0.893
Normal work	0.828	0.883
Recreational activities	0.840	0.881
Enjoyment of life	0.820	0.883

**Table 4:** PES-Ar test–retest reliability and measurement error (*n* = 50).

	Mean ± SD	ICC (2,1) (95%CI)	SEM	MDC95
PES-AR-Test	14.07 ± 5.45	0.88 (0.85, 0.92)	2.00	5.54
PES-Ar-Retest	14.85 ± 6.13			

ICC: Interclass Correlation Coefficient (Two-way random model); SEM: standard error of measurement for agreement; MDC95: minimal detectable change with 95% confidence.

**Table 5:** Factor Loadings for PES Scale.

Items	Factor Loadings
Mood	0.693
Ability to walk or move around	0.771
Sleep	0.837
Normal work	0.891
Recreational activities	0.899
Enjoyment of life	0.887

Extraction Method: Principal Axis Factoring: Rotation: None (single-factor solution).

**Table 6:** Spearman’ s Correlation Coefficient between PES, MFIS, PHQ-9, SF-MPQ, MSIS-29 (*n* = 121).

Variable	Spearman’s r with PES	*p*-Value	[95% CI]
PHQ-9	0.677	<0.001	[0.57, 0.77]
SF-MPQ	0.586	<0.001	[0.46, 0.69]
MFIS	0.660	<0.001	[0.55, 0.75]
MSIS-29 (Psychological)	0.085	0.354	[−0.09, 0.26]
MSIS-29 (Physical)	0.188	0.039	[0.01, 0.36]

r: Spearman’s correlation coefficient; CI: confidence interval; PES: Pain Effect Scale; MSIS-29: Multiple Sclerosis Impact Scale; MFIS: Modified Fatigue Impact Scale; PHQ-9: Patient Health Questionnaire-9; SF-MPQ: Short-Form McGill Pain Questionnaire; Significant at *p*-value < 0.01.

## Data Availability

Due to privacy restrictions, the data presented in this study are available upon request from the corresponding authors.

## Abbreviations

The following abbreviations are used in this manuscript:

MS	Multiple Sclerosis
CNS	Central Nervous System
HRQoL	Health-Related Quality of Life
RR	Relapsing-Remitting
SP	Secondary-Progressive
PES	Pain Effects Scale
PES-Ar	Arabic Version of the Pain Effects Scale
SF-MPQ	Short-Form McGill Pain Questionnaire
PHQ-9	Patient Health Questionnaire-9
MFIS	Modified Fatigue Impact Scale
MSIS-29	Multiple Sclerosis Impact Scale-29
GROC	Global Rating of Change
MOCA	Montreal Cognitive Assessment
MSQLI	Multiple Sclerosis Quality of Life Inventory
ICC	Intraclass Correlation Coefficient
SEM	Standard Error of Measurement
MDC95	Minimal Detectable Change (95% CI)
CVI	Content Validity Index
I-CVI	Item-Level Content Validity Index
S-CVI	Scale-Level Content Validity Index
KS	Kolmogorov–Smirnov Test
IRB	Institutional Review Board
KKUH	King Khalid University Hospital

## Appendix A

**Table A1:** Scoring direction of outcome measures used for construct validity.

Instrument	Higher Score Indicates
PES-Ar (Pain Effects Scale—Arabic)	Greater pain interference with daily activities and participation
MSIS-29 Physical subscale	Greater physical impact of multiple sclerosis
MSIS-29 Psychological subscale	Greater psychological impact of multiple sclerosis
PHQ-9	More severe depressive symptoms
MFIS	Greater fatigue impact
SF-MPQ	Greater pain intensity

**Table A2:** The original English items of the Pain Effects Scale (PES), their Arabic translations (PES-Ar), and the rationale for all translation and wording modifications.

Original English Item	Initial Arabic Translation	Final Arabic Translation (PES-Ar)	Modification Made	Rationale
mood	الحالة المزاجية	حالتك المزاجية	Added the possessive pronoun “ك” (your mood)	To enhance the personal nature of the item, directly referring to the respondent’s mood, thereby increasing the clarity of self-assessment.
ability to walk or move around	القدرة على المشي أو التحرك	قدرتك على المشي أو التحرك	Added the possessive pronoun “ك” (your ability)	To enhance the personal nature of the item, directly referring to the respondent’s ability to walk or move around, thereby increasing the clarity of self-assessment.
sleep	النوم	نومك	Added the possessive pronoun “ك” (your sleep)	To enhance the personal nature of the item, directly referring to the respondent’s sleep, thereby increasing the clarity of self-assessment.
normal work (both outside your home and at home)	العمل الطبيعي (سواء خارج المنزل أو داخله)	عملك المعتاد (سواء من المنزل أو خارجه)	1. Changed “العمل الطبيعي” (normal work) to “عملك المعتاد” (your usual work). 2. Reordered “خارج المنزل أو داخله” (outside or inside the home) to “من المنزل أو خارجه” (from home or outside).	1. The word “المعتاد” (usual) is more culturally appropriate and better describes daily routine than “الطبيعي” (normal), which might carry judgmental connotations. 2. Reordering the phrase makes it more fluid and natural in Arabic.
recreational activities	الأنشطة الترفيهية	أنشطتك الترفيهية	Added the possessive pronoun “ك” (your activities)	To enhance the personal nature of the item, directly referring to the respondent’s recreational activities, thereby increasing the clarity of self-assessment.
enjoyment of life	الاستمتاع بالحياة	قدرتك على الاستمتاع بحياتك	Changed to ‘قدرتك على الاستمتاع بحياتك’ (your ability to enjoy your life) by adding the phrase ‘your ability’ and using the possessive form ‘your life’.	The item was rephrased to emphasize the individual’s ‘ability’ to enjoy life, making it more action-oriented and personally relevant. This modification enhances the clarity of the item in the Arabic context, ensuring that respondents focus on the functional impact of pain on their quality of life rather than a general abstract concept
